# Supplementary material for: Are Efficient-Dose Mixtures a Solution to Reduce Fungicide Load and Delay Evolution of Resistance? An Experimental Evolutionary Approach
Source: Microorganisms. 2021 Nov 10;9(11):2324. doi: 10.3390/microorganisms9112324 (PMC8622124; doi:10.3390/microorganisms9112324)

Table S1: Commercial mixtures and single-fungicide formulations used to control Septoria leaf blotch on wheat in France. For each commercial product, the composition, recommended rate and use are detailed. Percentages indicate the fraction of each AI used in the mixture, relative to the commercial product including the same AI used alone, and the total line is the equivalent amount of fungicide in a mixture. Letters *a* to *h* refer to the commercial products based on a single fungicide compound : *a :* Elatus plus, *b :* Thore, *c :* Rubric, *d :* Imtrex, Syrex, Fluxatop*, e :* Sirena, *f :* Eyetak, Prochloflash, Pro Plex 450, Faxer, Fujara, Saranta, Sporaz, Septoraz*, g : Joao, Protioline, h :* Illiade, Mystic Ew, Fezan, Colnago, Rivazon, Erasmus, Spekfree, Curzol, Ulysses. *i* : Fungicides mixtures are described using abbreviation described in the column Fungicide. Letters *j* to *q* refers to refer to the commercial products based on fungicide mixture : *j:* Aviator Xpro, Oceor Xpro, SDH2 Pro, *k :* Karosse Xpro, Skyway Xpro, *l :* Cavando, Korema, Korema Star, Osiris Star, Osiris Win, Epomet, *m :* Adexar, Tenax XM, SDH1, *n :* Librax, SDH-CO, *o :* Ceratavo era, Elatus era, Velogy era, *p* : Kestrel, Onnel, Piano, Prosaro, Prosafort, Prosatop, *q :* Ampera, Diams, Epopée, Galactica, Nebraska.

				**Use in mixture**	**Fungicides**[i]	1	Bx + P + T [k]	E + M [l]	F + E [m]	F + M [n]	P+ B [o]	P+ T [p]	T + Pc [q]
					**Composition g.L^{-1}**	75+150	75+100+100	56.25+41.25 (or 37.5+27.5)	62.5+62.5	62.5+45	150+75	160+80 (or 125+125)	132.5+267.1
					**Recommended rate l.ha^{-1}**	1.25	1	2 (or 3)	2	2	1	1	1.5
**Use as a single compound**													
**Fungicide**	**Chemical class**	**Composition g.L^{-1}**	**Recommended rate l.ha^{-1}**	**Recommended use g.ha^{-1}**									
Benzovindiflupyr (B)[a]		100	0.75	75							100%		
Bixafen (Bx)[b]		125	1	125		75%	60%						
Epoxiconazole (E)[c]	SDHI	83	1.5	124.5				90%	100%				
Fluxapyroxad (F)[d]		62.5	2	125					100%	100%			
Metconazole (M)[e]	DMI	90	1	90				92%		100%			

Prochloraz (Pc)[f]		450	1	450									89%
Prothioconazole (P)[g]		250	0.8	200		94%	50%				75%	80% (or 62.5%)	
Tebuconazole (T)[h]		430 (or 250)	0.6 (or 1)	258 (or 250)			39-40%					31-32% (or 48-50%)	77-79.5%
					**Total**	**169%**	**149-150%**	**182%**	**200%**	**200%**	**175%**	**111-112% (or 110.5-112.5)**	**166-168.5%**

Figure S1: Correlation between susceptibilities to test fungicides in droplet tests.

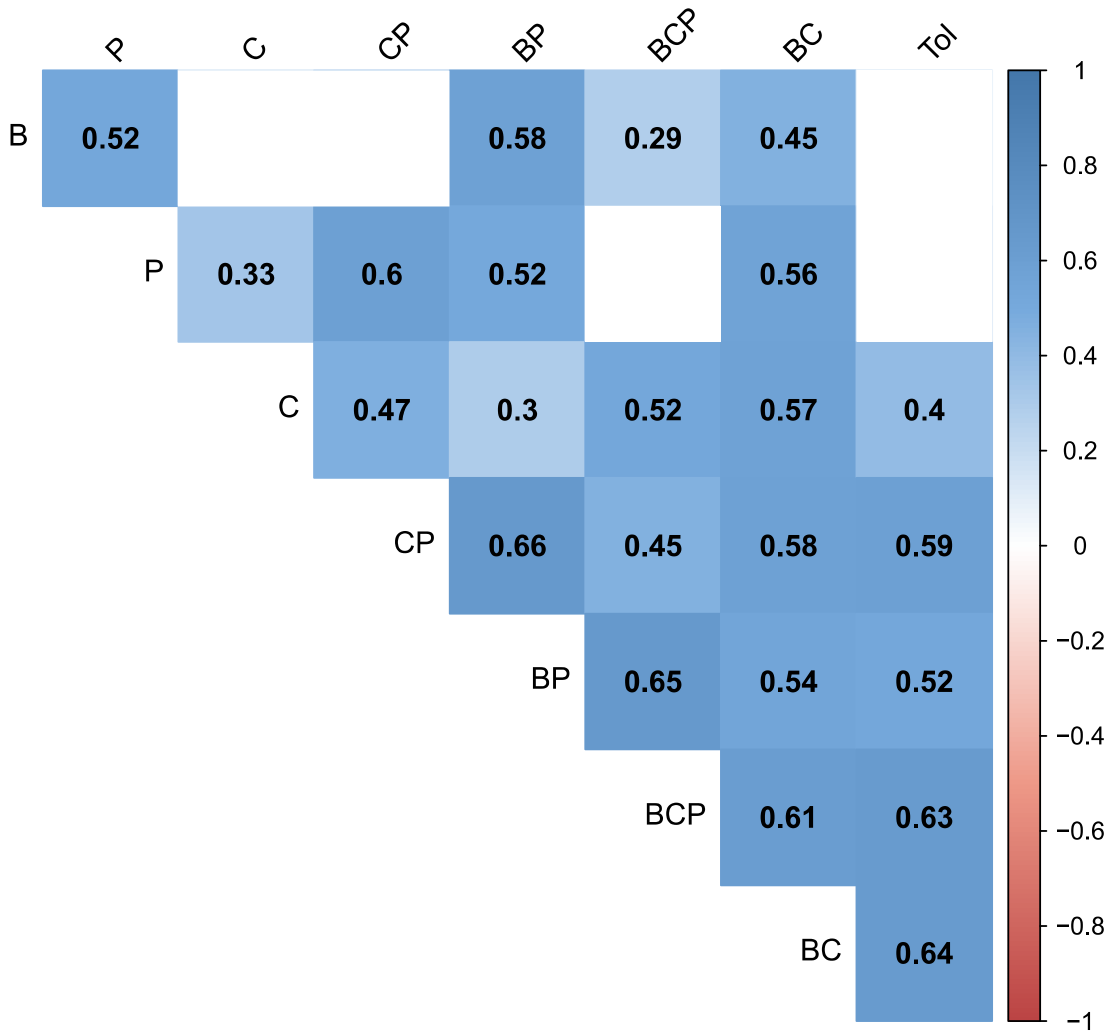

Supplement: Supplementary file 1 [file microorganisms-09-02324-s001.zip › microorganisms-1425136-supplementary.pdf]
